# Minimal differences observed when comparing the morphological profiling of microglia obtained by confocal laser scanning and optical sectioning microscopy

**DOI:** 10.3389/fnana.2024.1507140

**Published:** 2025-01-03

**Authors:** Sânziana Godeanu, Mădălina Iuliana Mușat, Anja Scheller, Eugen Osiac, Bogdan Cătălin

**Affiliations:** ^1^Experimental Research Centre for Normal and Pathological Aging, University of Medicine and Pharmacy of Craiova, Craiova, Romania; ^2^Department of Molecular Physiology, Center for Integrative Physiology and Molecular Medicine (CIPMM), University of Saarland, Saarbrücken, Germany; ^3^Center for Gender-Specific Biology and Medicine (CGBM), University of Saarland, Saarbrücken, Germany; ^4^Department of Biophysics, University of Medicine and Pharmacy of Craiova, Craiova, Romania; ^5^Department of Physiology, University of Medicine and Pharmacy of Craiova, Craiova, Romania

**Keywords:** microglia, cortex, morphology, optical sectioning microscope, confocal laser scanning microscopy

## Abstract

**Background:**

While widefield microscopy has long been constrained by out-of-focus scattering, advancements have generated a solution in the form of confocal laser scanning microscopy (cLSM) and optical sectioning microscopy using structured illumination (OSM). In this study, we aim to investigate, using microglia branching, if cLSM and OSM can produce images with comparable morphological characteristics.

**Results:**

By imaging the somatosensory microglia from a tissue slice of a 3-week-old mouse and establishing morphological parameters that characterizes the microglial branching pattern, we were able to show that there is no difference in total length of the branch tree, number of branches, mean branch length and number of primary to terminal branches. We did find that area-based parameters such as mean occupied area and mean surveillance area were bigger in cLSM isolated microglia compared to OSM ones. Additionally, by investigating the difference in acquisition time between techniques and personal costs we were able to establish that the amortization could be made in 6.11 ± 2.93 years in the case of countries with a Human Development Index (HDI) = 7–9 and 7.06 ± 3.13 years, respectably, for countries with HDI < 7. As such, OSM systems seem a valid option if one just wants basic histological evaluation, and cLSM should be considered for groups that demand higher resolution or volumetric images.

## 1 Introduction

Studying microglia in the central nervous system (CNS) is crucial in our understanding of the etiology and progression of neuroinflammatory diseases (Cătălin et al., 2013; Cojocaru et al., 2021). While direct electrophysiological and morphological studies of microglia are difficult, due to how fast microglia are reacting to their environment (Cătălin et al., 2017; Boboc et al., 2023), the link between microglial function and morphology allows researchers to accurately predict microglial involvement in such diseases (Hermann and Gunzer, 2020; Popova et al., 2021; Cornell et al., 2022). However, microglia exhibit a remarkable capacity for morphological plasticity, continuously surveilling their microenvironment through dynamic extension and retraction of cellular processes. The continuous morphological changes serve as a fundamental mechanism underlying their diverse functions, including synaptic pruning (Harry, 2013; Kettenmann et al., 2013; Arcuri et al., 2017; Weinhard et al., 2018), immune surveillance (Davalos et al., 2005; Nimmerjahn et al., 2005) and modulation of neuroinflammatory responses (Surugiu et al., 2019; Anton et al., 2021; Li et al., 2021). Different microglia phenotypes have been associated with both aging and disease, highlighting the importance of studying morphological changes as potential biomarkers for disease progression. Advancements in imaging techniques, such as laser scanning microscopy (LSM) have enabled detailed visualization and analysis of microglia morphology *in vitro* (Mitran et al., 2018; Stopper et al., 2018) and *in vivo* (Davalos et al., 2005; Nimmerjahn et al., 2005; Cătălin et al., 2013). These technological innovations offer unprecedented opportunities to unravel the intricate dynamics of microglial morphology in health and disease. Using such technologies, scientists have shown that microglia can change their morphology up to 8 h after death (Nimmerjahn et al., 2005; Block et al., 2007; Hanisch and Kettenmann, 2007; Ransohoff and Perry, 2009; Dibaj et al., 2010), and that as little as 5 min of global ischemia can be enough for microglia morphology change (Cătălin et al., 2017). Innovation in scientific research, particularly in the domains of imaging and microscopy, has historically been a catalyst for transformative discoveries. Central to the progress of microscopy is the quest for enhanced resolution, a critical parameter defining the performance of imaging systems. While traditional fluorescence microscopy has long been constrained by diffraction-limited resolution, recent strides have led to the emergence of super-resolution techniques capable of achieving resolutions at the nanoscale (Hell, 2007; Montgomery et al., 2013; Montgomery et al., 2016). However, all these advances pose a significant limitation: the high costs associated with acquiring them. While the total cost of confocal LSM (cLSM) has decreased, the level of the investment can still be restrictive for low-income countries. The introduction of optical sectioning microscopy using structured illumination (OSM) has emerged as a transformative imaging technique capable of providing high-resolution, three-dimensional visualization of biological specimens. While the base of OSM is widefield microscopy, by placing a grid between the sample and the detector to generate a pattern of intensity differences, the method can filter out-of-focus and by repetitive grid movements a true optical section is calculated (Neil et al., 1997). Although a direct comparison between the two methods has been made (Weigel et al., 2009), in the present study we aim to investigate if OSM can be used to investigate microglia morphology, and try to establish, using the difference in acquisition time and work force costs, a limit in which it does and does not become economically viable to acquire such devices compared to a cLSM system.

## 2 Materials and methods

### 2.1 Experimental animals

**Figure 1:**
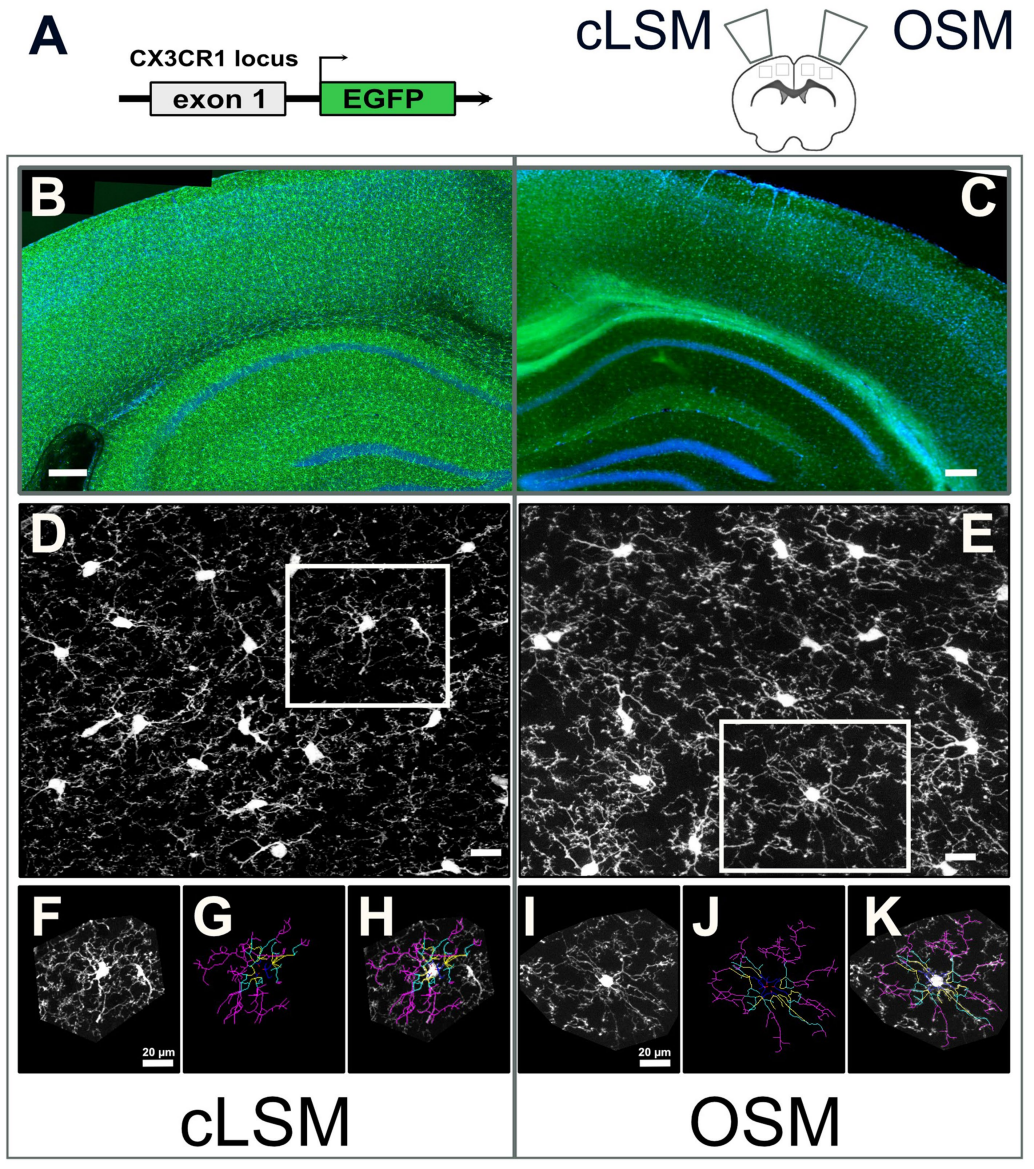
Schematic overview of techniques. **(A)** Construct of transgenic mouse line used in the present experiment with eGFP expressing microglia and random hemisphere assignment to cLSM or OSM acquisition. Example of processed overview of eGFP microglia expressing cells (green) and DAPI (blue) in the brain where **(B)** 20x stitching was used for cLSM or **(C)** 10x stitching was used for OSM (data used for exemplification, never used in the analysis of the images). **(D)** cLSM and **(E)** examples of eGFP microglia (white squares) (images used for analysis) where both soma and fine processes can be seen. Every cell included in the present study was isolated **(F,I)**, manually traced **(G,J)** and verified **(H,K)**. The scale bars in **(B,C)** indicate 250 and in **(D,E)** 20 μm.

All procedures were done on heterozygous transgenic TgH (CX_3_CR_1_-EGFP) mice (*n* = 4) (Figure 1A). After the genotype was confirmed (C57BL/6 N background) the procedures were done at 3 weeks of age (housing of animals was done in individually ventilated cages, on a 12-h (h) light/dark cycle at 20°C with both water and food ad libitum). All animal procedures were conducted at the animal facility of CIPMM, University of Saarland according to European and German guidelines for the welfare of experimental animals and approved by the Saarland “Landesamt für Gesundheit und Verbraucherschutz” in Saarbrücken/Germany (animal license number: perfusion2020).

### 2.2 Tissue preparation and image acquisition

After intraperitoneal anesthesia (ketamine 100 mg/kg; xylazine 10 mg/kg (Ketaset Zoetis, Parsippany-Troy Hills Township USA; Rompun, Bayer Vital Leverkusen, Germany)), animals were subjected to trans-cardiac perfusion with phosphate-buffered saline (PBS) (ThermoScientific, 10010023) followed by 4% paraformaldehyde (ThermoScientific, 30525-89-4) (PFA, pH 7.4 in PBS pH 7.4, 0.1 M). Brains were kept overnight 4% PFA at 4°C, as recommended in order to ensure the least amount of microglia activation (Cătălin et al., 2017). Brain slices (35 μm) were prepared as coronal sections using a Leica VT1000S vibratome (Leica Biosystems, Wetzlar, Germany), stained with DAPI (25 ng/mL) (Fluoromount-G with DAPI, ThermoScientific, 00–4,959-52), mounted and sealed on microscopy slides.

Image stacks were acquired with two different types of microscopes: OSM (ApoTome, Axio Imager.Z2) and the Confocal Microscope (LSM 880, Axio Observer, both Zeiss, Oberkochen, Germany) (Figures 1B,C). For this study microglia of the somatosensory cortex were sampled (Figures 1D,E). We randomly assigned either the right or left cortex to be sampled using the OSM or the cLSM. For both methods, a standardized scanning protocol was used to acquire similar sized images (Table 1).

**Table 1:** Technical information and settings regarding image acquisition.

Microscope	ApoTome (Axio Imager.Z2)	Confocal microscope (LSM 880, Axio Observer)
Z-stack	12 μm	15 μm
Scaling (per Pixel)	0.161 μm/0.161 μm/1.00 μm	0.42 μm/0.42 μm/1.00 μm
Image Size (scaled)	223.82 μm/167.70 μm	212.55 μm/212.55 μm
Objective	EC Plan-Neofluar 40x/0,75 M27	Plan-Apochromat 40x/1.3 Oil DIC UV-IR M27
Excitation wavelength/Source	488 nm / HXP 120 V	488 nm / Diode laser 10 mW,
Filters used (Ex./Em.)	450-490 nm / 500–550 nm	400–568 nm
Exposure Time	400 ms	394.40 ms
Pixel time	2.76 μs	1.54 μs
Detection	AxioCam MR R3	GsAsP-PMT

All images were acquired using the Zeiss software ZEN 3.2 (ZEN lite).

### 2.3 Image analysis

In order to quantify microglial morphology, a semi-manual method was used that was previously applied in quantifying microglia morphology (De Lucia et al., 2016; Liu et al., 2021) and other glial populations (Braun et al., 2015; Di Benedetto et al., 2016; Strat et al., 2022). Briefly, from each full Z-stack, microglia with complete arborization were manually isolated. In order to ensure a comparable arborization, only cells with the body in the centre of the z stack were used. The analysed maximum projection of each cell was represented by collapsing 5 μm above and below the centre of the target microglia (Godeanu et al., 2023) (Figures 1F,I). Microglia with processes that extend outside the stack limits were not included in the present study. All images were processed using Zen Software (Carl Zeiss, Jena, Germany) and Fiji (Meijering et al., 2004; Šimunić et al., 2024). For each animal, 10 cells were isolated (5 cells scanned using OSM and 5 contralateral cells scanned with cLSM). After skeletisation (Figures 1G,H,J,K), for each cell the total length of the branch tree, the number of branches, the mean branch length and the number of each branch order were used to compare the two methods. Additionally, the occupied area and the area surveyed by each cell were also analysed.

### 2.4 Cost-efficiency analysis

In order to correctly assess the investment opportunity in one system or another, the approximate system cost, the acquisition cost of one pixel and the average researcher’s salary were gathered through an online search. The obtained data was grouped according to the country’s Human Development Index (HDI) (Table 2).

**Table 2:** Randomly selected countries are divided in terms of HDI.

HDI > 9			HDI = 7–9			HDI < 7		
Country	Salary (USD/year)	Years	Country	Salary (USD/year)	Years	Country	Salary (USD/year)	Years
Australia	76,115	1.31	Belarus	7,970	12.54	Brasil	19,083	5.24
Austria	74,436	1.34	Bulgaria	14,901	6.71	India	9,209	10.85
Canada	87,165	1.14	China	26,069	3.83	Indonesia	20,579	4.85
Denmark	57,816	1.72	Estonia	25,605	3.90	Irak	17,272	5.78
Germany	68,041	1.46	Hungary	19,589	5.10	Mexico	15,966	6.26
New Zeeland	61,884	1.61	Polonia	25,673	3.89	Peru	12,674	7.89
Norway	76,874	1.30	Romania	19,051	5.24	Pakistan	7,032	14.22
Sweden	64,118	1.55	Russion Federation	15,561	6.42	South Afrika	22,201	4.50
United Kingdom	52,982	1.88	Slovakia	26,992	3.70	Uruguay	19,963	5.00
United States	77,284	1.29	Turkey	10,249	9.75	Vietnam	16,604	6.02

Three groups have been defined as HDI < 7, HDI = (7–9) and HDI > 9 (10 countries per group). For each country the mean annual researcher salary (USD/year) and the time, measured in years, represents the duration over which the researcher’s salary could be covered by the price difference between the two microscopes. Currency units other than USD were converted as per the exchange rate at that particular moment. Countries are displayed in alphabetical order.

### 2.5 Data analysis

An average value for each parameter was introduced in Graph-Pad Prism 9.3 and/or Microsoft Excel. After confirming the normal distribution using the Kolmogorov–Smirnov test, an unpaired *t*-test was performed individual for each analyzed parameter. In all figures the mean and standard deviation (SD) are displayed. Individual data are displayed in the form of small points (individual microglial measurement) and large points (surrounded by circles- representing the mean obtained for each animal). Statistical significance is depicted as follows: *p < 0.05, **p < 0.01, and ***p < 0.001.

## 3 Results

### 3.1 Minimal difference in basic microglia morphology can be observed between techniques

**Figure 2:**
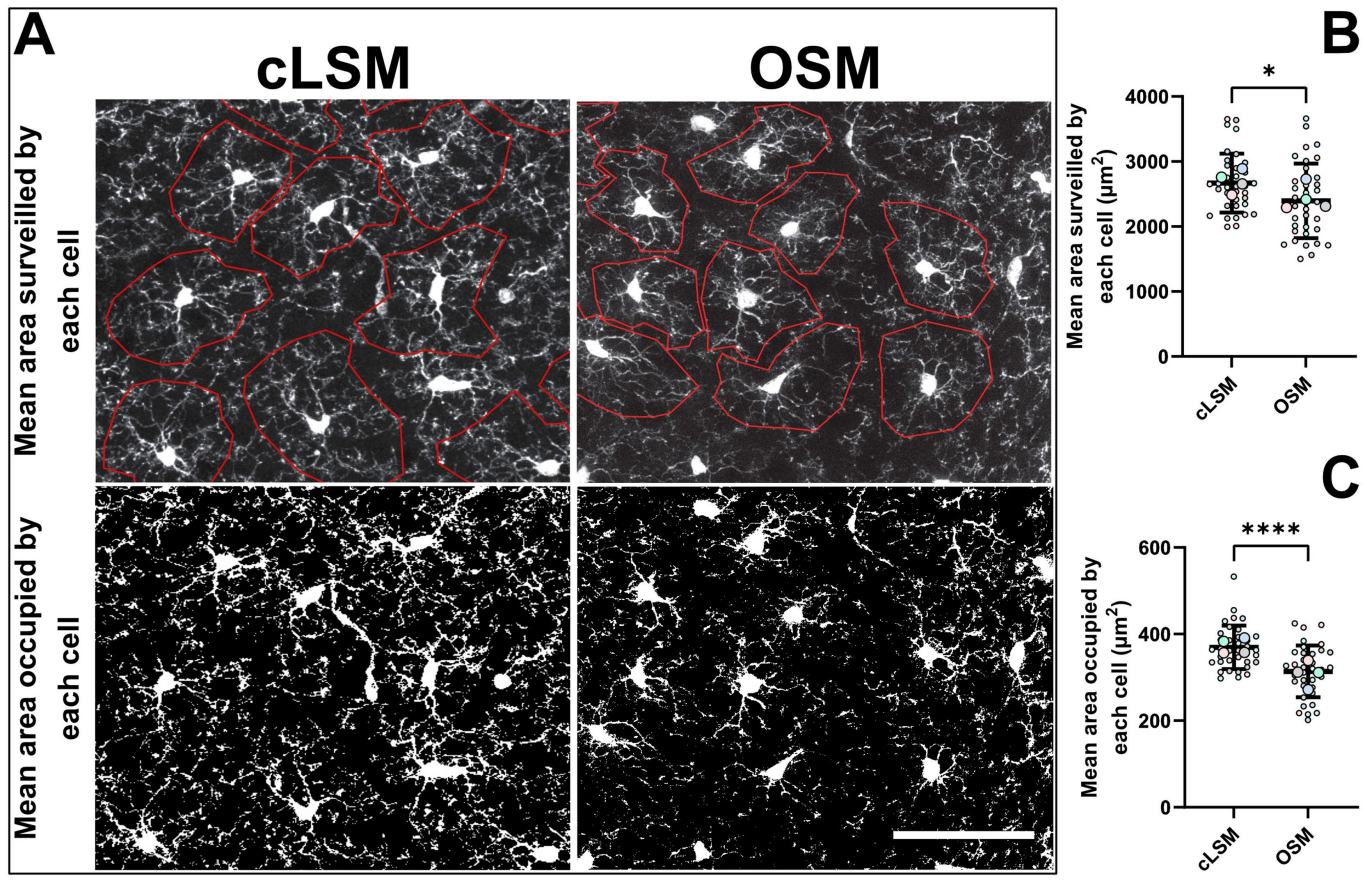
Differences in area-based parameters. **(A)** Examples of microglia obtained by cLSM and OSM acquisition in which the mean cell surveilled area was determined. Area based parameters were the only differences obtained between cLSM and OSM when analyzing microglia morphology. **(B)** The average area occupied by microglia was 314 ± 59.71 μm^2^ when determined by the OSM and 369.40 ± 50.37 μm^2^ when microglia obtained by cLSM were analyzed (*p* < 0.0001). **(C)** The mean surveilled area was lower using the OSM method (2395 ± 574 μm^2^) compared to cLSM (2669 ± 453 μm^2^) (*p* = 0.03). The scale bars indicate 100 μm.

**Figure 3:**
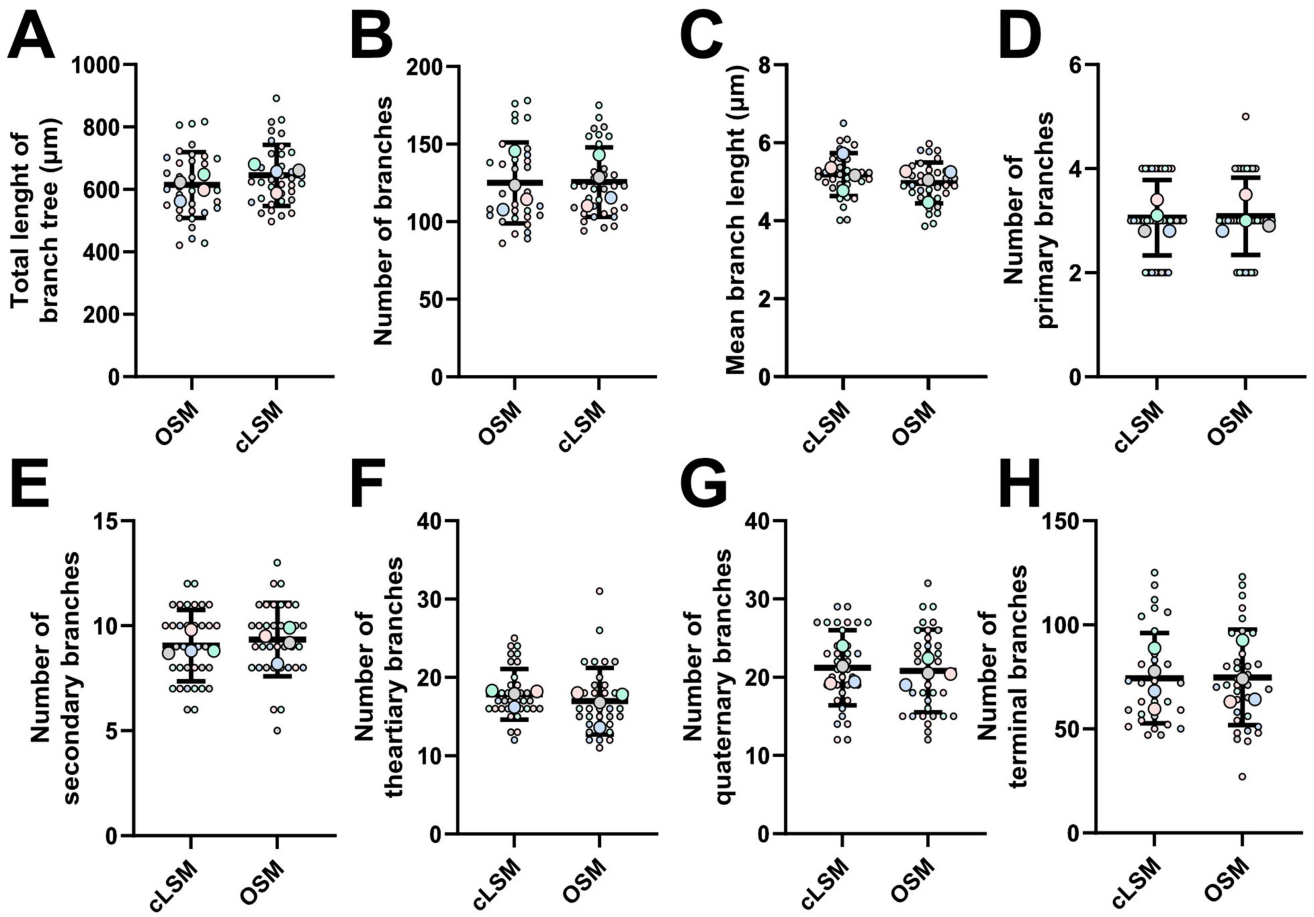
Microglia arbor morphology is comparable between cLSM and OSM acquisitions. By tracking each branch, we were able to demine detailed microglia morphology starting with basic parameters as **(A)** the total arbor length, **(B)** number of and **(C)** mean branch length. None of these parameters showed differences between the two methods. Additional detailed morphological analysis was also unable to show differences between the number of **(D)** primary, **(E)** secondary, **(F)** tertiary, **(G)** quaternary and **(H)** terminal branches. Each colored larger circle represents an animal, and the same-colored dots represent the analyzed cells from that animal.

Applying basic microglia measurements revealed no difference between the two methods. But by measuring area-based parameters, we were able to detect some differences (Figure 2). As such, both the mean area occupied by a cell and the mean surveilled area were lower using the OSM method compared to cLSM (Figures 2A,B). The mean surveilled area of microglia cells acquired with cLSM was 2669 ± 453 μm^2^ compared to 2395 ± 574 μm^2^ in OSM (*p* = 0.03) (Figure 2A). The difference was higher when comparing area occupied by microglia using the OSM (314 ± 59.71 μm^2^) and cLSM (369.40 ± 50.37 μm^2^) (*p* < 0.0001) (Figure 2B). When exploring the individual morphological parameters of the branching pattern, by converting the microglia into a topological skeleton depiction, no significant differences between our two data sets were found (Table 3). A large arborization was detected for the mice using both methods. Using the OSM a total branch length of 607.70 ± 36.50 μm was determined as compared to 646.60 ± 40.40 μm obtained by cLSM acquisition (*p* = 0.2) (Figure 3A). The OSM method discriminated, on average, 125 ± 26.15 branches compared to 125.60 ± 22.41 branches obtained using the cLSM (*p* = 0.922) (Figure 3B), with a mean branch length of 5.18 ± 0.55 μm determined by cLSM compared to 4.97 ± 0.52 μm for the OSM acquisition (*p* = 0.095) (Figure 3C).

**Table 3:** Results of morphological analysis and differences obtained with the unpaired *T*-test for parameters used in the morphological analyses, when comparing the cLSM and OSM.

Unpaired *T*-test					
		cLSM (Mean ± SD)	OSM (Mean ± SD)	R square	*p*-value
Mean occupied area		369.40 ± 50.37	314.00 ± 59.71	0.2060	<0.0001
Mean surveillance area		2669.00 ± 453.00	2395.00 ± 574.60	0.0673	0.0300
Total length		645.20 ± 97.70	614.30 ± 105.70	0.0231	0.2085
Number of branches		125.60 ± 22.41	125.00 ± 26.15	0.0001	0.9221
Mean branch length		5.18 ± 0.55	4.97 ± 0.52	0.0402	0.0958
Number of branches for each order	Primary	3.05 ± 0.72	3.08 ± 0.74	0.0003	0.8711
	Secondary	9.05 ± 1.69	9.34 ± 1.74	0.0070	0.4901
	Tertiary	17.86 ± 3.22	16.97 ± 4.23	0.1364	0.3679
	Quaternary	21.23 ± 4.80	20.80 ± 5.26	0.0018	0.7232
	Terminals	74.34 ± 21.70	74.74 ± 22.97	<0.0001	0.9405

To provide a detailed morphological quantification, we classified each branch within a cellular tree as determined by both imaging techniques (Figures 3D–H). The mean number of primary microglia branches was similar between the two techniques, with 3.06 ± 0.74 for the OSM and 3.05 ± 0.72 for cLSM (Figure 3D) (*p* = 0.871). A similar result was also observed when investigating the average number of secondary branches, with the OMS being able to discriminate 9.34 ± 1.74 branches compared to the 9.05 ± 1.69 as determined by cLSM (Figure 3E) (*p* = 0.49). The semi-manual technique used, generated similar results between OSM scanned microglia and cLSM ones when determining the number of tertiary and quaternary, with OSM scanned microglia having on average 16.97 ± 4.23 tertiary branches and 20.80 ± 5.26 quaternary ones compared to 17.86 ± 3.22 tertiary (*p* = 0.32), respectively 21.23 ± 4.80 quaternary ones (*p* = 0.72) (Figures 3F,G). Determining terminal branches using both methods yielded also similar results, with OSM being able to discriminate 74.74 ± 22.97 branches compared to 74.43 ± 21.70 as determined by cLSM (*p* = 0.94) (Figure 3H) (Table 3).

### 3.2 Cost amortization between the techniques differs around the world

**Figure 4:**
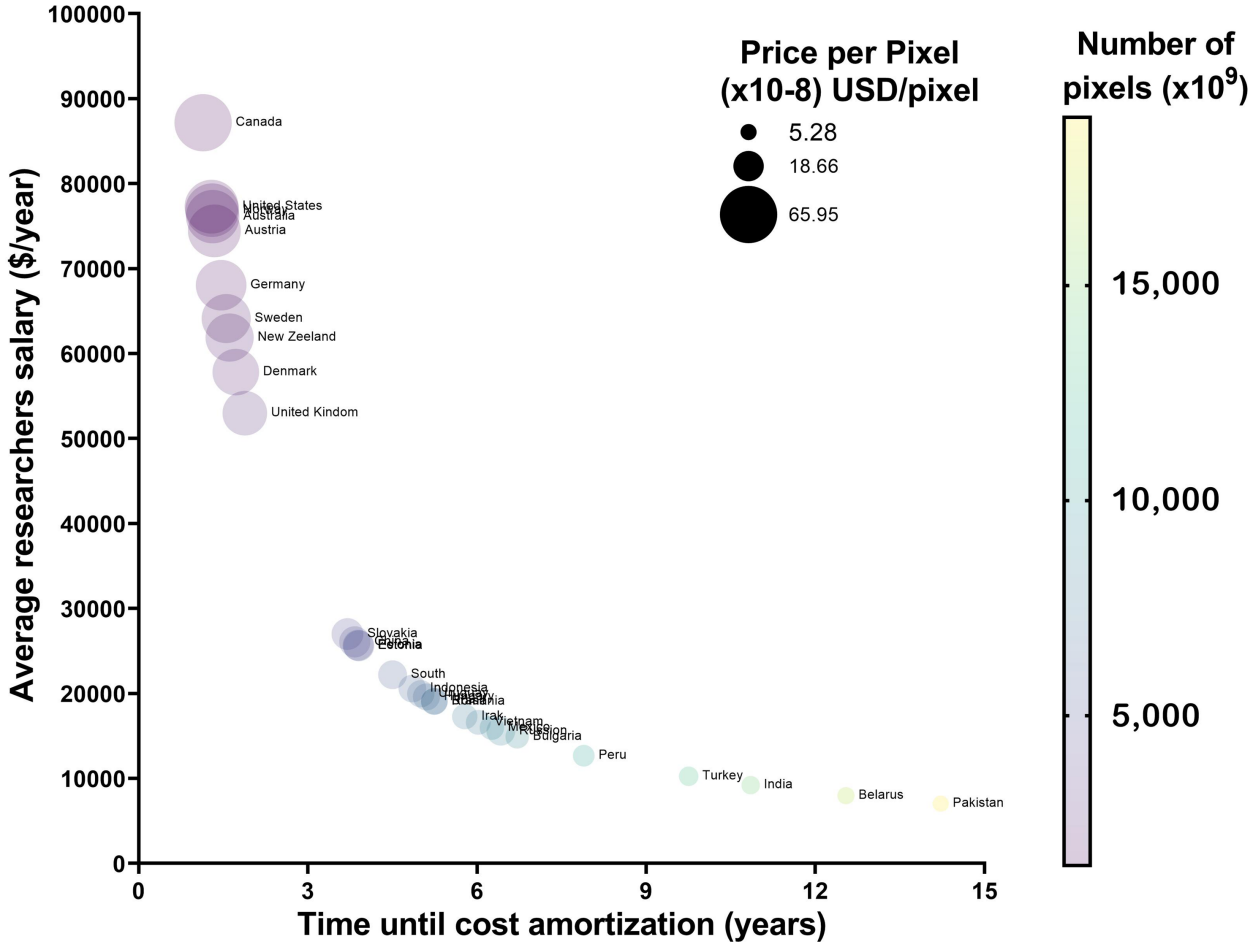
Cost amortization in randomly selected countries. Countries with a HDI over 9 will not benefit from acquiring OSM systems, as the long acquisition time per pixel will mean that the total number of pixels until parity will be under 5×10^12^. This is opposed to countries where HDI is under 7 and the personal costs are lower and as such there is a longer time in which one can use the system before reaching the point of parity in pixel acquisition.

Given the minimal differences in microglial morphology between OSM and cLSM described above, and the difference in the initial price of the two methods, we wanted to investigate what would be the number of pixels acquired or the years needed to justify the initial investment, taking into account the average salary of a hypothetical researcher using one or the other method (Table 2). Across the sampled countries, it would take approximately 4.87 ± 3.40 years or 6,486 ± 4597×10^9^ pixel at a cost of 26.53 ± 19.62×10^−8^ USD in order to justify the difference in cost. However, this is not the same for all sampled countries, as countries with a Human Development Index (HDI) > 9 have only between 1.14 to 1.88 years for countries like Canada of the UK. Countries with a HDI between 7 and 9 have on average around 5 years to balance the two costs. However, for countries with an HDI < 7 the initial investment in a cLSM system seems hard to justify, as the difference between cLSM and OSM systems will only be balanced after 12.54 years in countries such as Belarus and 14.22 years in Pakistan (Figure 4).

## 4 Discussion

Because light microscopy is considered a fundamental tool in most areas of life science research, its use in any research laboratory seems like a given. From the use of glass lenses to the optical systems of today’s microscopes, researchers can choose from a great variety of techniques including: confocal microscopy (Minsky, 1988), STED microscopy (Hell and Wichmann, 1994; Okada and Nakagawa, 2015), Adaptive Optics (AO) z-STED Microscopy (Barbotin et al., 2019), Spinning Disc Confocal Microscopy (SPDM) (Oreopoulos et al., 2014), Airyscan Microscopy (Wu and Hammer, 2021) or OSM (ApoTome, Zeiss) (Ryan et al., 2017; Tröger et al., 2020). As with most technologies, the initial cost is high, but as production and competition begins, the overall market drives the price down. However, due to economic disparities around the world (Holstein et al., 2009; Peterson, 2017; Lee, 2023) the affordability of a product varies (Lipsey and Swedenborg, 2007). In contrast, access to information is getting easier. For example, a group of researchers in any country connected to the Internet can access the latest results in any known field in seconds. Thus, while curiosity and knowledge may be present in low-income labs, lacking infrastructure may prevent testing different theories. The introduction of new methods that use simple ideas can lead to surprising results, from an increase in diagnosis of certain diseases (Bhamla et al., 2017) to every day house comforts (Graettinger et al., 2005). While there are major differences between state-of-the-art techniques and cheaper alternatives, in certain situations and for certain questions it is just not economically viable (and necessary) to opt for the top-of-the-line solution.

Here, we could show that advances in OSM research make this technique a suitable, albeit time-consuming, alternative for cLSM. While the image quality of the two microscopic techniques is influenced by several factors, including the type of sample, the expertise of the team, and the requirement for identifying cellular or subcellular structures, by utilizing the same tissue sample and the same experienced team proficient in analyzing morphological parameters of microglia, we have mitigated some of those variables. Thus, the major differing factor lies in the technical disparities between the two microscopes. Microglia were chosen both because our laboratory has already gained experience with microglia morphology (Cătălin et al., 2017) and because of the high morphological variability of microglia (Godeanu et al., 2023). We also chose to investigate the microglia from the somatosensitive cortex of 3-week-old mice, as they were shown to have a complex branching pattern, compared to microglia obtained from aged animals (Godeanu et al., 2023; Pinosanu et al., 2023), hoping that differences between the two techniques would become apparent using this complex cell.

To date, resolution comparisons between the two microscopy techniques have primarily been performed within customized setups (Jonkman and Brown, 2015). Due to our experience in brain imaging and glial cells (Cătălin et al., 2017), we chose to investigate the somatosensory cortical microglial cells for their morphological appearance as dynamic and highly ramified cells under physiological conditions (Davalos et al., 2005; Nimmerjahn et al., 2005; Garaschuk and Verkhratsky, 2019; Lier et al., 2021). The microglial processes in our study were traced and labeled starting with primary branches (the first order branches that arise from the soma) and terminal branches (that are the most distant from the cell body located ramifications). No differences were seen in the analyzed morphological parameters between our two groups, showing that OSM can be a viable alternative to cLSM in certain morphological studies.

cLSM was the only technique that allowed for the rejection of out-of-focus light (Weigel et al., 2009). The development of OSM (which does not require an excitation source or pixel-by-pixel scanning of the object) has demonstrated the ability to achieve a resolution that, according to previous studies, can potentially surpass that of cLSM (Gustafsson, 2000) and has proven its usefulness with remarkable diagnostic results (Das et al., 2012; Heintzmann and Huser, 2017). As such, choosing the best method, between the two, in order to answer a biological question boils down to potential artifacts that accompany OSM. Although the quality of images generated by both methods can be affected by photobleaching or vibration-induced artifacts, OSM may provide better images from very weak photo-samples due to detection device (CCD camera instead of photomultiplier) but is additionally affected by artifacts induced by light scattering in thicker samples (≥30 μm) (Weigel et al., 2009). As a result, image quality degrades with increasing tissue depth because the grid pattern is projected onto the focal plane, which is contaminated by scattered light (Weigel et al., 2009). In the present study we used image stacks of approximately 15 μm acquired from a 35 μm slice. Therefore, a possible reason for the comparable results between the two techniques may be that the depth of our acquisition is insufficient for such artifacts to manifest, or at least to significantly affect the overall results. Moreover, other factors were shown to impact the image quality of OSM. For example, noise can be quantified as information, and the final image can thus become corrupted. This was shown to be the case for image noise picked up by OSM, especially starting with depths more than z = 27 μm, due to increasingly low contrast (Weigel et al., 2009). This low contrast, resulting from the increased noise, leads to a fragmented view of thin structures, which can cause a false reduction in branch numbers, especially in a manual analysis approach.

We found smaller average area-based parameters obtained by OSM compared to cLSM. With microglia being highly dynamic and their surveilled area always shifting due to microglia constantly emitting and retracting their processes with the highest-order extensions being the most mobile (Cătălin et al., 2017), one might argue that the difference between the two methods can be a consequence of the measured populations. However, the present reported mean surveilled area and mean occupied area obtained with the cLSM were comparable to our previous reports (*p* = 0.1314, respectively *p* = 0.3469 Supplementary Figure 1). As discussed, OSM imaging scattering is greater as the depth of the tissue is increasing. The bright fluorescence emitted by the soma or first-order branches can produce so much noise, that it can be counted as information, resulting in a larger false value when evaluating the exact edge of the surveillance area, for example. With a difference of approximately 10% between the average surveillance area determined by OSM and cLSM, for the resolution of the analyzed pictures, the difference in choosing the edge is around 2 μm (Supplementary Figure 2). Furthermore, it is possible that we have incorporated a z averaging effect on our investigations. As mentioned in the image acquisition and analysis’s part z-stacks were used for analyses and as proven structured illumination methods can generate enhanced optical sectioning compared to confocal methods (Wilson, 2011). These differences in optical narrowing of sectioning intensity can be as much as 20–25%, which may be one reason for a reduced fluorescence signal. In addition, structured illumination methods (such as Apotome) are not suitable methods for the study of living moving specimens due to the technical limitations of having to acquire three images at three different positions of the Ronchi rule for each z, which can increase scattering. Furthermore, the difference in resolution between the two methods generated by different objectives and specific machine bound parameters can also add to the observed changes.

While both techniques have advantages and disadvantages that the investigator needs to be aware of, another important aspect that should be taken into consideration is the financial one. With the average initial considerable investment difference between an OSM and a cLSM system (approximately 100,000 USD), planning the questions that need answering, seems to be just one aspect in deciding between the two methods. Although today’s society is evolving exponentially in terms of discoveries made, research and scientific results are still dominated by the resources a particular laboratory has at its disposal (Ioannidis and Garber, 2011), major improvements can be made “on a budget” (Sendi et al., 2004). Beyond the initial acquisition cost, the ongoing financial burden of routine maintenance, servicing, and repairs of advanced instruments poses a substantial challenge. This is particularly pronounced in less developed countries, where the availability of technical expertise and replacement parts may be limited, leading to increased costs. In addition, funding and grants are highly dependent on other factors such as country’s economic status and although the differences between developed and developing countries have narrowed in the 21st century, they remain or are unevenly distributed, including in the field of research (Paprotny, 2021). For example, a study that analyzed the biomedical publications from several developed and emerging countries (1994–2013), showed that even tough countries like Malaysia are catching up with some developed countries in terms of the number of publications, there are still large gaps. Moreover, the percentage of gross domestic product (GDP) spent on research is lower, especially when comparing the United States (2.7% of their GDP in 2011) to Malaysia (1.06%) or Qatar (0.33%) (Tang et al., 2016). Hence, based on our findings, allocating funding smartly (considering outcome and labor cost), particularly in the context of developing countries, may be more beneficial than investing it in state-of-the-art fast microscopy systems. Thus 100,000 USD could cover the salary of a researcher for 6.11 ± 2.93 years in the case of countries with an HDI = (7–9) and for 7.06 ± 3.13 years for HDI < 7 (Figure 4) (Table 2).

## 5 Conclusion

Since there are no significant differences in the results provided by the morphological analysis done for this study, with area-based parameters being the exception, we can presume that for this scope and other basic histological evaluation, OSM systems like the ApoTome could be an appropriate choice given a reduced initial budget and a lower labor cost. For projects that require fast moving *in vivo* samples, more detailed analyses or focus on a volumetric image or an increased resolution one should consider cLSM systems as the first choice.

## Data Availability

The raw data supporting the conclusions of this article will be made available by the authors, without undue reservation.

## Supplementary material

Supplementary Figure 1 Comparison between area-based parameters between the current set of data and previous reported ones. **(A)** Mean area occupied by each cell, although higher on the current study (372.20±17.72 μm^2^) compared to previous reports (280.85±101.98 μm^2^) the difference did not reach statistical difference *p* = 0.1294. **(B)** The same trend can be seen for the mean area surveilled by each cell.

Supplementary Figure 2 Example of five random OSM isolated microglia. **(A)** For just a 10% increase in surveilled area (pixelated area around the isolated cells) the total **(B)** difference in length added is under 2 μm. This distance can be seen as smaller averages in length determined parameters as seen in Figure 3.
