# Supplementary figures and images for: Minimal differences observed when comparing the morphological profiling of microglia obtained by confocal laser scanning and optical sectioning microscopy
Source: Front Neuroanat. 2025 Jan 3;18:1507140. doi: 10.3389/fnana.2024.1507140 (PMC11739110; doi:10.3389/fnana.2024.1507140)

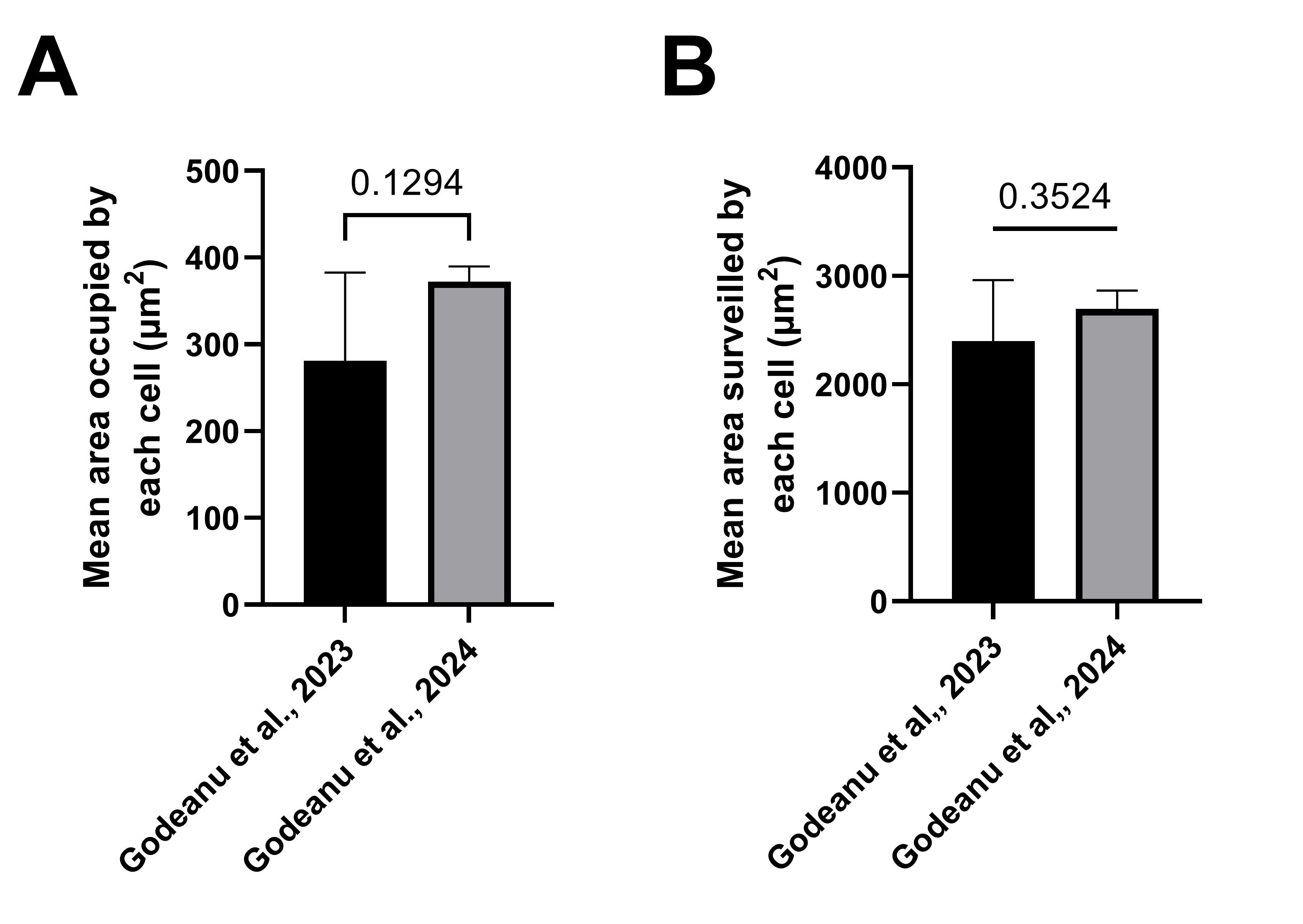

Supplement: Supplementary Figure 1 — Comparison between area-based parameters between the current set of data and previous reported ones. (A) Mean area occupied by each cell, although higher on the current study (372.20±17.72 μm2) compared to previous reports (280.85±101.98 μm2) the difference did not reach statistical difference p = 0.1294. (B) The same trend can be seen for the mean area surveilled by each cell. [file Image_1.JPEG]

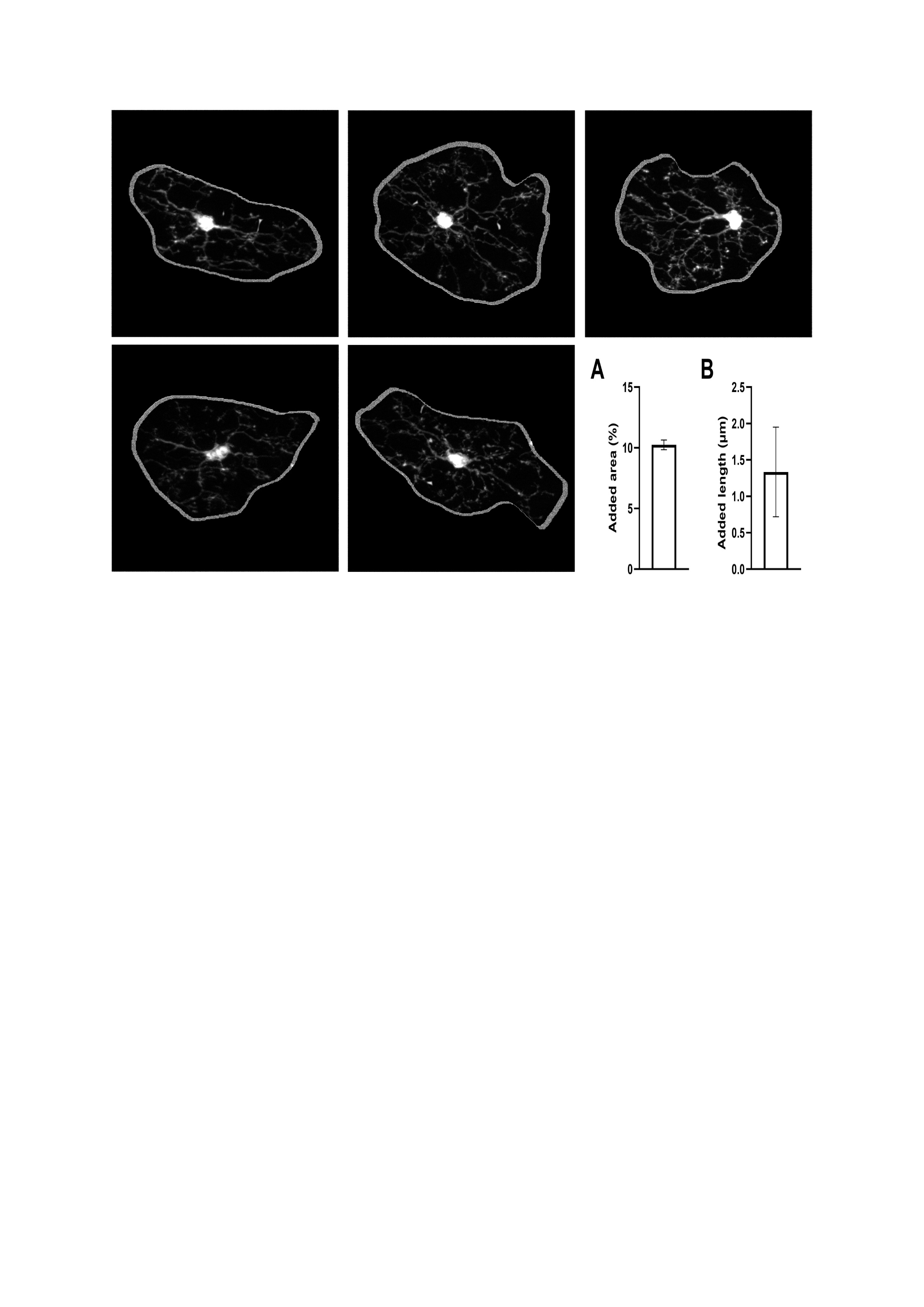

Supplement: Supplementary Figure 2 — Example of five random OSM isolated microglia. (A) For just a 10% increase in surveilled area (pixelated area around the isolated cells) the total (B) difference in length added is under 2 μm. This distance can be seen as smaller averages in length determined parameters as seen in Figure 3. [file Image_2.jpg]
